# Pharmacological Preconditioning with Diazoxide Upregulates HCN4 Channels in the Sinoatrial Node of Adult Rat Cardiomyocytes

**DOI:** 10.3390/ijms26136062

**Published:** 2025-06-24

**Authors:** Wilibaldo Orea, Elba D. Carrillo, Ascención Hernández, Rubén Moreno, María C. García, Jorge A. Sánchez

**Affiliations:** Department of Pharmacology, Center for Research and Advanced Studies of the National Polytechnic Institute, Mexico City 07360, Mexico; wilibaldo.orea@cinvestav.mx (W.O.); elcarrillo@cinvestav.mx (E.D.C.); ashernandez@cinvestav.mx (A.H.); vete_173@hotmail.com (R.M.); cgarcia@cinvestav.mx (M.C.G.)

**Keywords:** sinoatrial node cells, preconditioning, HCN4 channel, reactive oxygen species, c-Fos

## Abstract

Cardioprotection against ischemia is achieved using openers of mitochondrial ATP-sensitive K^+^ (mitoKATP) channels such as diazoxide (DZX), leading to pharmacological preconditioning (PPC). We previously reported that PPC decreases the abundance of ventricular Cav1.2 channels, but PPC’s effects on other channels remain largely unexplored. In this study, we hypothesized that DZX regulates the expression of hyperpolarization-activated cyclic nucleotide potassium channel 4 (HCN4) channels in sinoatrial node cells (SANCs), the specialized cardiomyocytes that generate the heartbeat. DZX increased the heart rate in intact adult rats. Patch-clamp experiments revealed an increase in the magnitude of ionic currents through HCN4 channels, which was abolished by the reactive oxygen species (ROS) scavenger N-acetylcysteine (NAC) and the selective mitoKATP channel inhibitor 5-hydroxydecanoate (5-HD). Quantitative reverse transcriptase polymerase chain reaction (qRT-PCR) and Western blot assays showed that DZX increased HCN4 channel expression at the mRNA and protein levels. Immunofluorescence analyses revealed that PPC increased HCN4 fluorescence, which was abolished by NAC. DZX increased nuclear translocation of c-Fos and decreased protein abundance of RE1 silencing transcription factor (REST)/neuron-restrictive silencer factor (NRSF), suggesting the involvement of these factors. Our results suggest that PPC increases the heart rate by upregulating HCN4 channel expression through a mechanism involving c-Fos, REST, and ROS.

## 1. Introduction

Damage to cardiac muscle caused by ischemia can be largely prevented by preceding brief periods of ischemia [1] or pharmacological agents such as DZX [2,3,4]. Both forms of preconditioning depend on the opening of mitoKATP channels which increases ROS generation in mitochondria, thereby precluding mitochondrial Ca^2+^ overload and mitochondrial permeability transition pore opening [5,6,7]. Although mitoKATP channels are crucial, other ion channels related to Ca^2+^ homeostasis, such as Cav1.2 and Orai channels, also play relevant roles during the preconditioning of ventricular cardiomyocytes [4,8,9]. However, possible changes in the activity or expression of other channels in the heart chambers have remained largely unexplored.

The rhythmic activity of the heart depends on the sinoatrial node consisting of specialized myocytes that fire spontaneous action potentials. This pacemaker activity has been attributed to the activation of membrane ion channels, but critically timed Ca^2+^ releases also play a role [10]. Ion channel activity underlying pacemaker activity in sinoatrial node cells (SANCs) has been studied extensively [11], and vast experimental evidence indicates that HCN4 channels are crucial players. HCN4 channels are activated by the negative membrane potential occurring during the diastolic phase, producing the “funny current” or If [12,13]. There are two main types of sinoatrial myocytes, both of which express HCN4 channels: the relatively small (~40 µm long) spindle cells typically located in the central region of the sinoatrial node and elongated (~80 µm long) cells, usually located in the periphery [14,15,16].

In this study, we examined the effects of PPC with DZX on the heart rate in intact rats, on HCN4 channel currents recorded in spindle and elongated cells, on the mRNA and protein expression of HCN4 channels in SANCs, and on the abundance and intracellular localization of the transcription factors c-Fos and REST/NRSF. We used the patch-clamp technique to record whole cell membrane currents, Western blotting and qRT-PCR to quantify mRNA and protein levels in the presence of various drugs, with the aim of elucidating the mechanisms involved. We also examined whether ROS are involved in the upregulation of the HCN4 protein by DZX using the ROS scavenger NAC, and tested the possibility that changes in the expression of HCN4 channels are accompanied by changes in the expression or cellular localization of REST and c-Fos using Western blotting and immunofluorescence.

## 2. Results

### 2.1. PPC Increased the Heart Rate and HCN4 Channel Currents

**Figure 1:**
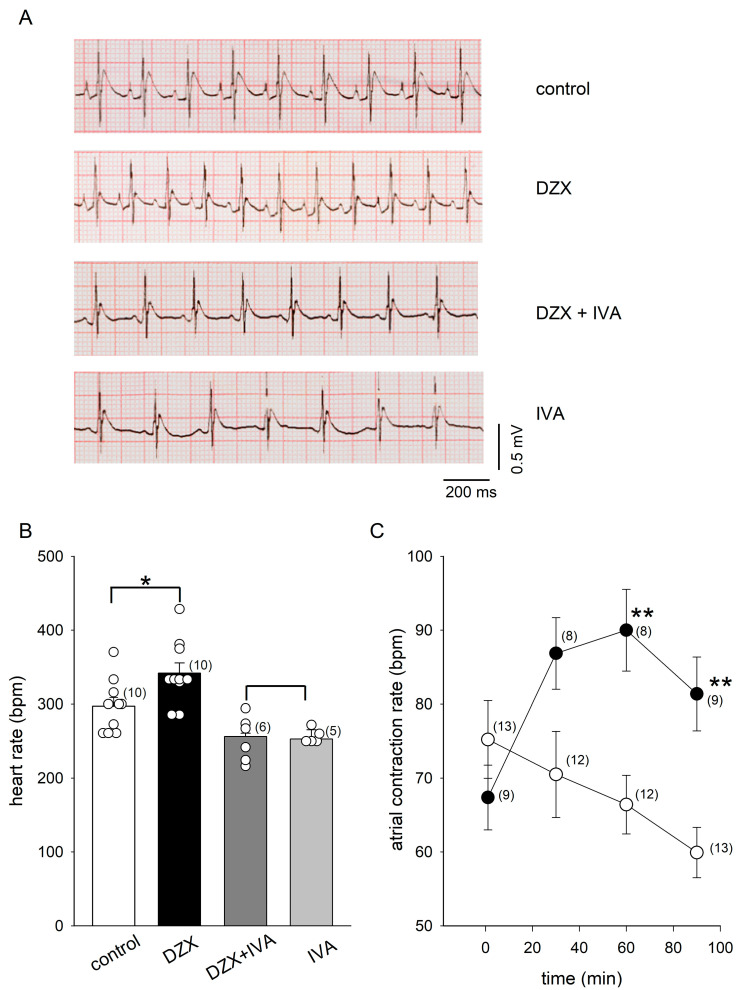
Effects of DZX on the heart rate. (**A**) Representative ECG recordings obtained in rats under the indicated experimental conditions. (**B**) Mean (±SEM) heart rates from the same experiments as in (**A**). Each symbol represents a separate experiment. (**C**) Mean (±SEM) contraction rates of isolated atria under control conditions (open circles) and after incubation in DZX (black circles). In (**B**,**C**), the numbers of experiments are indicated in parentheses. * *p* < 0.05, ** *p* < 0.01. IVA, ivabradine.

We found that DZX increased the heart rate in rats (Figure 1A,B). The HCN4 blocker ivabradine decreased the heart rate by itself, as expected, and completely suppressed the effect of DZX, suggesting that HCN4 channels play a role in the response to DZX (Figure 1A,B). Consistent with these results, DZX also increased the isolated atrial contraction rate (Figure 1C).

**Figure 2:**
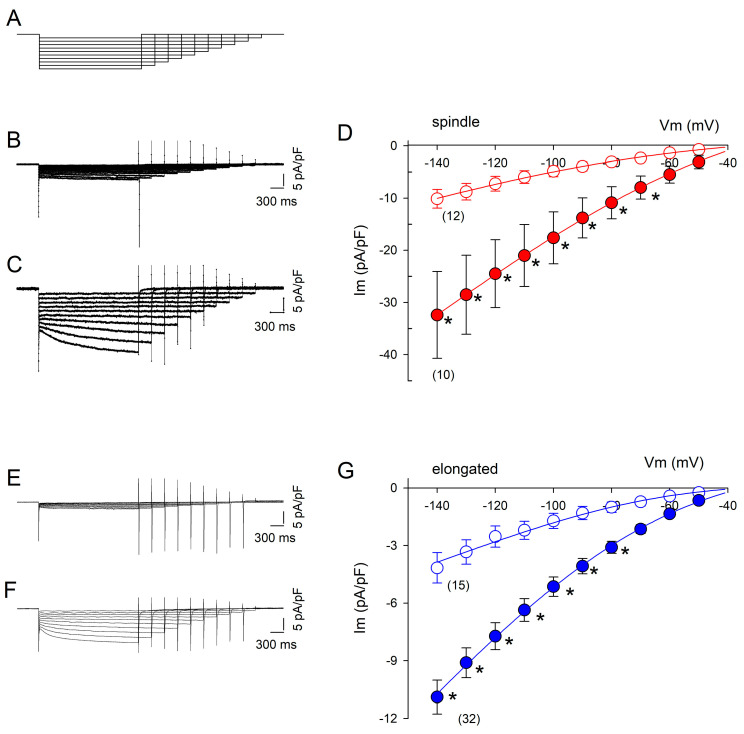
Effects of DZX on the If current amplitude. (**A**) Pulse protocol. (**B**,**C**) Representative recordings of membrane currents (Im) from spindle cells under control and DZX treatment conditions, respectively. (**D**) Mean (±SEM) current–voltage relationships of If currents from spindle cells from the same experiments as in (**B**,**C**) under control (open circles) and DZX treatment (red circles) conditions. Solid lines are best fits of Equation (3) to average values, with gf = 0.13 nS/pF and 0.36 nS/pF, respectively. (**E**,**F**) Representative recordings from elongated cells under control conditions and after DZX treatment, respectively. (**G**) Mean (± SEM) current–voltage relationships of If currents from elongated cells from the same experiments as in (**E**,**F**) under control (open circles) and DZX treatment (blue circles) conditions. Solid lines are the best fits of Equation (3) to average experimental values, with gf = 0.04 nS/pF and gf = 0.13 nS/pF, respectively. In (**D**,**G**), the numbers of experiments are indicated in parentheses. * *p* < 0.05.

The results of the patch-clamp experiments performed in the whole-cell configuration to measure If currents are summarized in Figure 2. DZX greatly increased the If current magnitude in spindle and elongated cells. The If current amplitude increased significantly during hyperpolarizing pulses. The If values were larger for spindle cells than for elongated cells under control and DZX treatment conditions. In both cell types, DZX increased gf values by approximately three times. It is important to point out that changes in If current amplitude were only observed in preconditioned cells requiring a prolonged preincubation time in DZX and that DZX by itself had no significant effects on If magnitude. The ratio between the amplitude of the If current recorded at −140 mV under control conditions relative to the value recorded 2–3 min after DZX application averaged 1.07 ± 0.04 (SEM) in four independent experiments.

**Figure 3:**
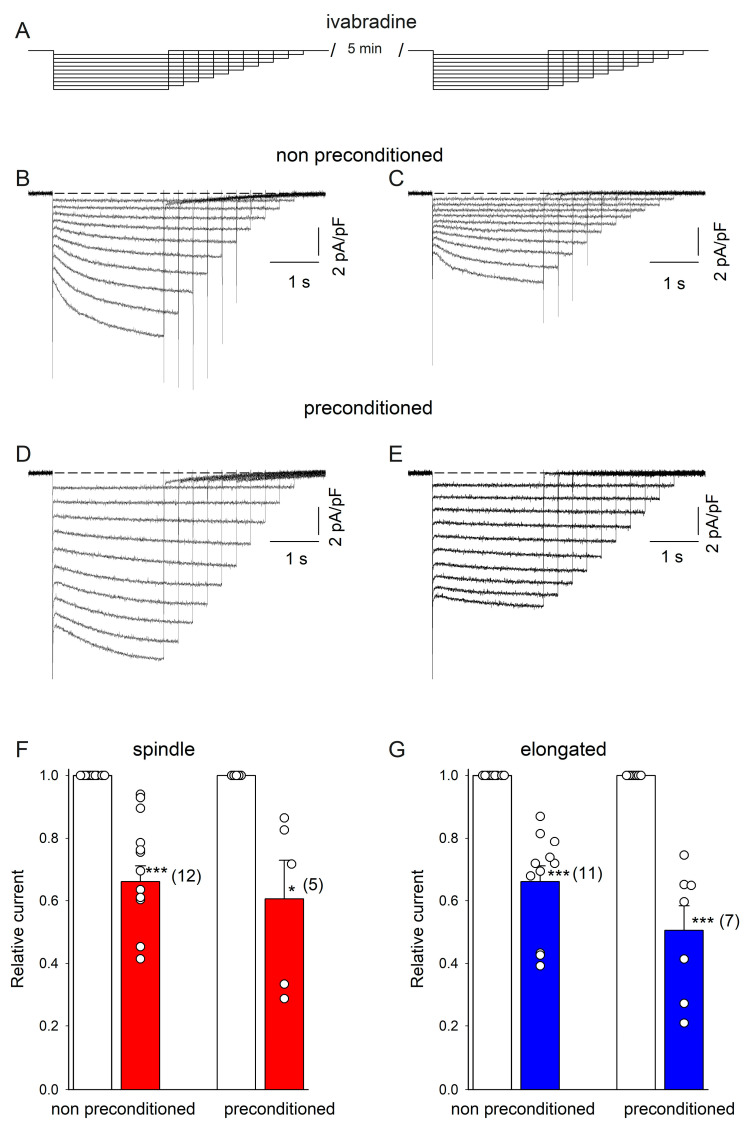
Action of ivabradine on If currents in control and DZX-preconditioned cells. (**A**) Experimental protocol. (**B**,**C**) Representative HCN4 currents from a control spindle cell before and after ivabradine treatment conditions, respectively. (**D**,**E**), Representative HCN4 currents from a DZX-treated spindle cell before and after ivabradine application, respectively. Dashed lines represent the current value of 0. (**F**,**G**) Mean (±SEM) relative current values (post-ivabradine/pre-ivabradine) at the ends of –140 mV hyperpolarizing pulses in control and DZX-treated spindle and elongated cells, respectively. Each symbol represents a separate experiment, and the numbers of experiments are indicated in parentheses. * *p* < 0.05, *** *p* < 0.001.

Next, we used the HCN4 channel blocker ivabradine to partially block HCN4 channel currents. We hypothesized that if the increase in current amplitude by DZX is due to larger HCN4 currents, a similar reduction in the relative current amplitude by ivabradine would be expected in both DZX-treated and control cells. First, SANCs were recorded in the absence of ivabradine and then ivabradine was added to the bath solution and currents were recorded again in the same cells. Consistently, ivabradine reduced membrane currents similarly in DZX-pretreated and control SANCs of both types (Figure 3), implicating HCN4 currents.

### 2.2. ROS Are Involved in PPC

**Figure 4:**
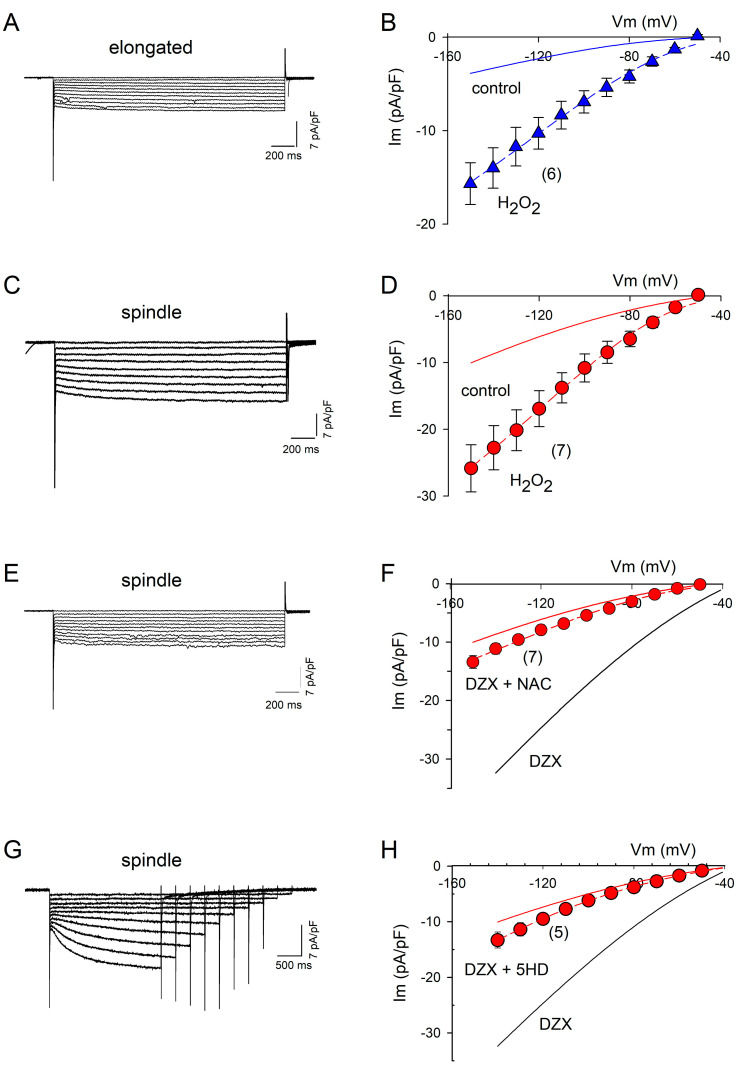
Action of ROS on If current amplitudes. (**A**) Representative recordings of If at several potentials from an elongated cell after the addition of H_2_O_2_ to the bath solution. (**B**) Mean (±SEM) I–V relationships from several experiments. The dashed line represents the best fit of Equation (3) to mean If current values, with gf = 0.14 nS/pF. The solid line represents the best fit of Equation (3) to the control If currents from elongated cells shown in Figure 2. (**C**) Representative recordings of If at several potentials from a spindle cell after the addition of H_2_O_2_ to the bath solution. (**D**) Mean (±SEM) I–V relationships from several experiments. The dashed line represents the best fit of Equation (3) to mean If current values, with gf = 0.24 pS/nF. The solid line represents the best fit of Equation (3) to If from control spindle cells shown in Figure 2. (**E**,**G**) Representative recordings of If currents at several potentials from experiments performed with spindle cells incubated with DZX plus NAC or 5-hydroxydecanoate. (**F**,**H**) Mean (±SEM) current–voltage relationships of If currents from spindle cells under the conditions shown in (**E**,**G**). Dashed lines represent best fits of Equation (3) to mean currents, with gf = 0.11 pS/pF and gf = 0.16 pS/pF, respectively. Red solid lines are best fits of Equation (3) to average If values under control conditions, and black solid lines are best fits to the average If currents from DZX-treated spindle cells shown in Figure 2. The numbers of experiments are indicated in parentheses.

The addition of H_2_O_2_ to the bath solution in the electrophysiological experiments increased the If amplitude by approximately threefold relative to the control in spindle and elongated cells (Figure 4A–D), supporting a role of increased ROS production in the response to DZX. The ROS scavenger NAC and mitoKATP channel blocker 5-hydroxydecanoate abolished the effect of preconditioning on the If amplitude, confirming the role of ROS (Figure 4E–H).

### 2.3. DZX Increases HCN4 Channel Conductance

**Figure 5:**
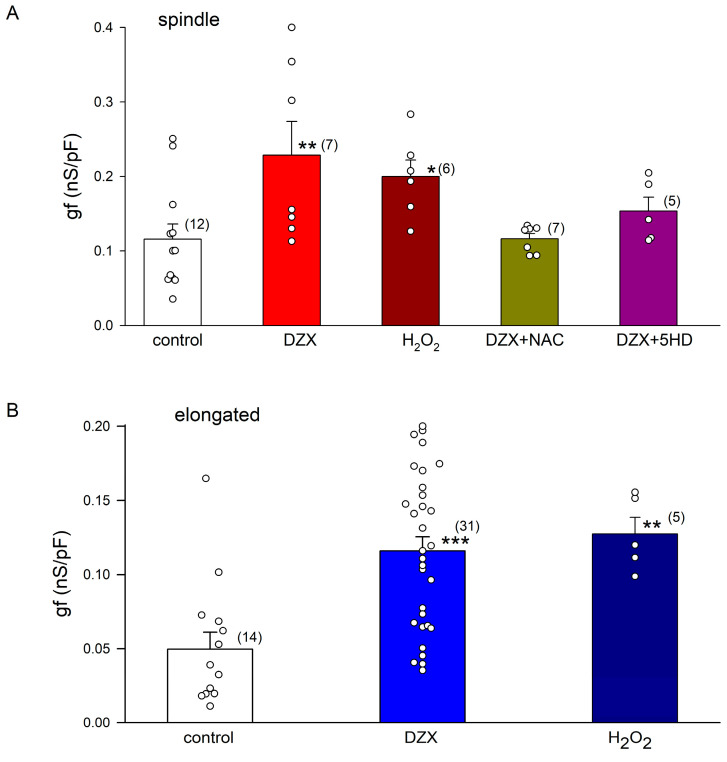
The effect of DZX on gf is mediated via ROS. Mean (± SEM) gf values from spindle (**A**) and elongated cells (**B**) under the indicated experimental conditions. Each symbol represents a separate experiment, and the number of cells is indicated in parentheses. * *p* < 0.05, ** *p* < 0.01, *** *p* < 0.001. Differences were assessed relative to control values.

Figure 5 summarizes the results of a quantitative analysis of the experiments shown in Figure 2 and Figure 4. Equation (3) was fitted to the If values of each current–voltage relationship under the indicated experimental conditions. Illustrated are mean values (±SEM) of the maximum conductance (gf). DZX and H_2_O_2_ significantly increased gf in spindle (Figure 5A) and elongated cells (Figure 5B). Consistent with the role of ROS on gf increase, NAC and 5-HD prevented the action of DZX in spindle cells (Figure 5A).

### 2.4. DZX Increased HCN4 Channel Expression

**Figure 6:**
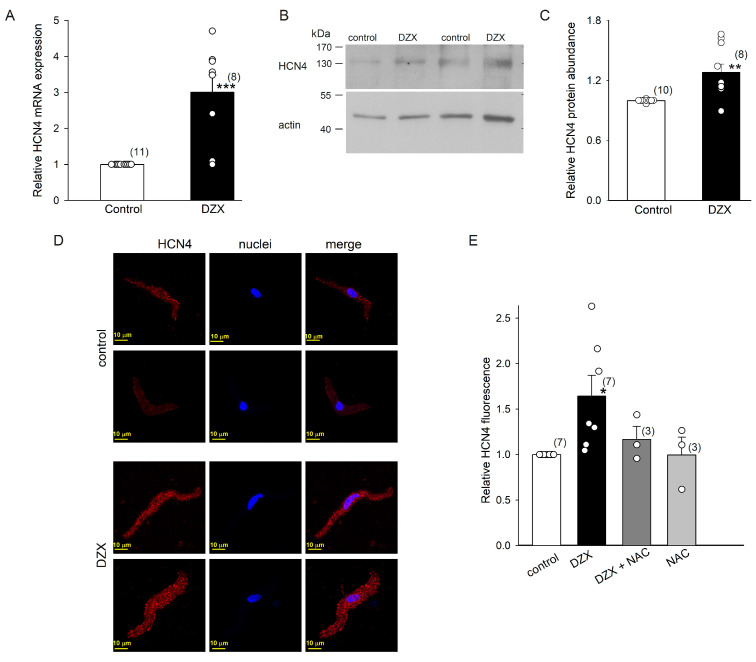
Effects of preconditioning with DZX on HCN4 mRNA and protein abundance. (**A**) Increase in relative HCN4 mRNA expression in SANCs after DZX treatment. (**B**) Representative blots of HCN4 from two separate experiments. (**C**) Relative HCN4 protein abundance in whole fractions of SANCs. Relative HCN4 density values were normalized using actin bands. (**D**) Representative SANC immunofluorescence under control conditions and after DZX treatment from two separate experiments. (**E**) Mean (±SEM) HCN4 fluorescence under the indicated conditions. Each symbol represents a separate experiment, and the numbers of experiments are indicated in parentheses. * *p* < 0.05, ** *p* < 0.01, *** *p* < 0.001.

DZX upregulated HCN4 channels at the mRNA and protein levels in SANCs, as confirmed by qRT-PCR experiments and Western blots (Figure 6A–C). Western blotting analysis showed that the HCN4 protein had a molecular mass of ~140 kDa, close to the expected molecular weight (132 kDa). These results indicate that an increase in the number of HCN4 channels explains the higher maximal conductance values observed in association with the DZX-induced increase in If amplitude. In agreement with these results, confocal microscopy revealed an increase in HCN4 fluorescence in DZX-treated SANCs (Figure 6D,E). This increase was abolished by the ROS scavenger NAC, in line with the electrophysiological results (Figure 4).

### 2.5. Effects of DZX on REST and c-Fos Transcription Factors

**Figure 7:**
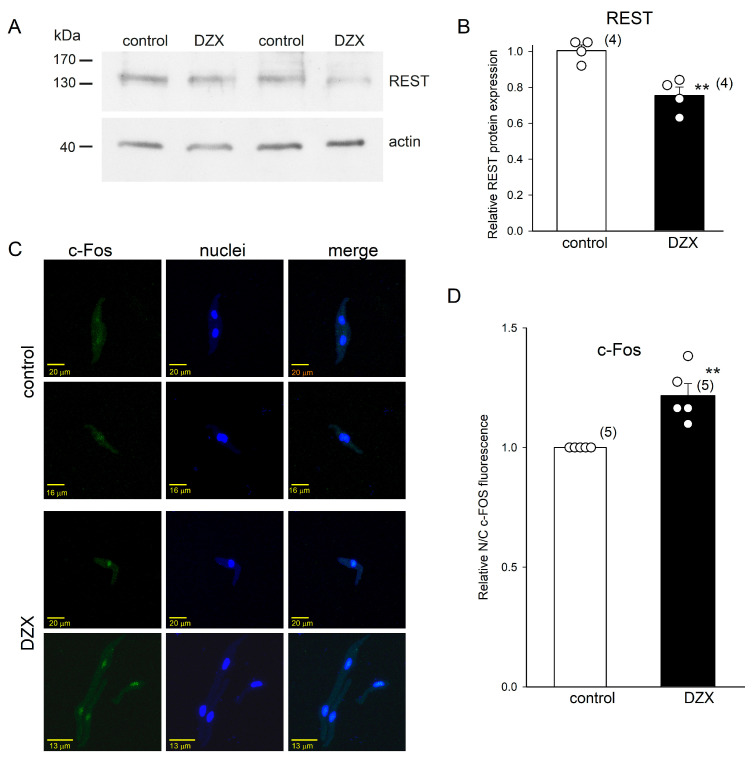
Effects of preconditioning with DZX on REST protein abundance and c-Fos localization. (**A**) Representative blots of REST from two separate experiments. (**B**) Relative REST protein abundance in whole fractions of SANCs. REST density values were normalized using actin bands. (**C**) Representative SANC immunofluorescence under control conditions and after DZX treatment from two separate experiments. (**D**) Mean (±SEM) c-Fos fluorescence under the indicated conditions. In (**B**–**D**), each symbol represents a separate experiment, and the numbers of experiments are indicated in parentheses. ** *p* < 0.01.

We performed Western blot and immunofluorescence experiments to test the hypothesis that transcription factors REST/NRSF and c-Fos play a role in the increase in HCN4 channel expression by DZX shown above. We found that DZX reduced the integrated densities of REST/NRSF protein bands (molecular mass slightly > 130 kDa), with an approximately 30% reduction in the relative protein abundance, in SANCs (Figure 7A,B). Immunofluorescence experiments showed that DZX induced an increase in the translocation of c-Fos to SANC nuclei (Figure 7C,D). These results indicate that these transcription factors may play a role in the increase in HCN4 channel expression by DZX.

## 3. Discussion

In this study, we made the novel observation that DZX increased the If amplitude in SANCs in a ROS-dependent manner. We further showed that DZX increased the abundance of HCN4 channels. The upregulation of the HCN4 protein was shown to depend on the de novo synthesis that followed changes in the expression of its mRNA. The rise in ROS production associated with ischemic preconditioning or PPC with drugs such as DZX plays a key role in protection against ischemia. ROS production increases as a result of mitoKATP channel opening [3,4,7,17,18,19], and we previously showed in experiments performed with NAC in ventricular cardiomyocytes that the protection conferred by ROS depends on downregulation of Cav1.2 channels [4]. In the present study, experiments performed with NAC and 5-hydroxydecanoate demonstrated the involvement of ROS generated by mitochondria in increments of the If amplitude and the associated membrane conductance. We also found that incubation with H_2_O_2_ greatly increased the If magnitude, further confirming the direct involvement of ROS. A much higher concentration of H_2_O_2_ (200 μM) had the opposite effect, moderately decreasing the If amplitude recorded at −100 mV, in rabbit SANCs [20]. Besides possible species differences, this discrepancy could be due to the deleterious effects of the high H_2_O_2_ concentration used by Guo et al. [20] on membrane currents. In addition, If values obtained at a single membrane potential may reflect slight shifts along the voltage axis in the current–voltage relationship and not changes in the maximum conductance of HCN4 channels.

Our experiments also demonstrated that PPC with DZX downregulated REST/NRSF in SANCs. REST/NRSF is a transcription factor that is crucially involved in the repression of neural genes and plays critical roles in non-neuronal cells such as cardiomyocytes [21]. Its repression depends on its binding to specific DNA RE1 motifs [22,23]. REST/NRSF strictly regulates the expression of HCN4 during cardiac muscle development [24,25,26,27], and a conserved REST/NRSF binding site in the promoter region of the HCN4 gene has been characterized [24]. The physiological regulation of HCN4 expression by REST/NRSF has also been observed in the adult heart. For example, in exercised rats, REST/NRSF upregulation leads to HCN4 downregulation and sinus bradycardia [28]. Given the importance of REST as a repressor of HCN4 expression, the DZX-induced reduction in REST/NRSF abundance observed in this study is likely involved in the upregulation of HCN4 channels and increase in If currents caused by PPC.

**Figure 8:**
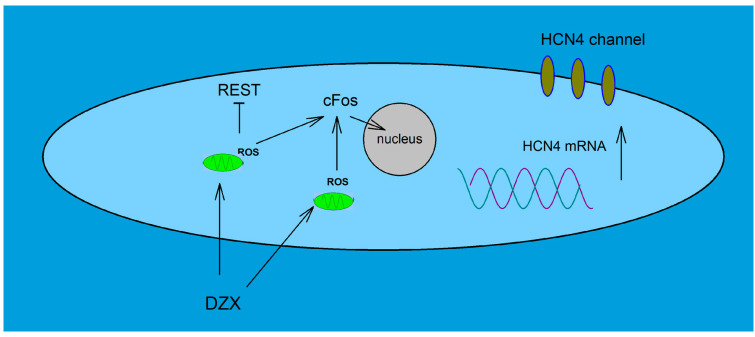
Role of PPC on HCN4 expression. Schematic showing hypothetical signaling mechanisms related to DZX actions on the HCN4 gene via ROS, c-Fos and REST on upregulation of HCN4 channels in SANCs.

We also observed that nuclear translocation of c-Fos increased by DZX in SANCs, as described previously in ventricular cardiomyocytes [9]. c-Fos is an early response gene and a component of activator protein 1 transcription factor (AP-1) [29]. It may be involved in the upregulation of HCN4 by DZX. The enhancer regions of non-coding sequences in the HCN4 gene locus have been found to contain highly conserved binding sites for AP-1, suggesting that this transcription factor plays an important role in HCN4 transcription [30]. In addition, it is interesting to note that AP-1 is responsive to ROS [31] and H_2_O_2_ treatment can induce rapid increases in c-Fos expression in cardiomyocytes [32]. It is plausible that c-Fos and REST/NRSF are involved in the coordinated regulation of HCN4 gene transcription, though the exact mechanism remains to be established. In previous work, three clusters of transcription initiation sites and three promoters in the 5′ end of the REST/NRSF gene, and several enhancer and repressor regions that may regulate REST/NRSF expression in non-neural cells were described and the promoter regions contain several putative AP-1 binding sites [33]. Thus, c-Fos, as a member of the AP-1 complex, may downregulate the expression of REST. This possibility is supported by the findings that c-Fos may repress gene transcription [34] and that c-Jun, another member of the AP-1 complex, represses REST transcription through AP-1 binding sites [35]. In conclusion, it is plausible that upregulation of HCN4 expression by PPC can be explained by increased translocation of c-Fos and downregulation of REST by ROS (Figure 8).

The upregulation of HCN4 channels by PPC is expected to increase the heart rate. DZX was previously observed to increase the heart rate [36], and our present experiments revealed that it induced higher rates of contraction in intact rats and isolated atria. These increases are likely related to an increase in the abundance of HCN4 channels, which are predominant among HCN channels in SANCs and play a key role in pace-making by generating If [37]. The increase in heart rate may have beneficial effects during preconditioning, increasing the cardiac output and thereby compensating for the decrease in stroke volume during each beat associated with smaller Ca^2+^ signals during action potentials due to a decrease in the Cav1.2 channel content [4]. A reduced ventricular Ca^2+^ transient amplitude during an action potential is expected to reduce the stroke volume during a single beat because the contraction force is related closely to pCa in cardiac muscle [38]. Smaller Ca^2+^ signals during action potentials were also observed in SANCs after PPC with DZX (Figure S1).

## 4. Materials and Methods

### 4.1. Animals

Male adult (7–8 weeks old) Wistar rats, weighing 185–200 g were used in this study. Rats were obtained from the Animal Production and Experimentation Unit (UPEAL) of the Center for Research and Advanced Studies of the National Polytechnic Institute (UPEAL–CINVESTAV–IPN; Mexico City, Mexico). They were housed four per cage in a room maintained at 21–24 °C with a 12:12 h light–dark cycle and fed standard rat chow.

### 4.2. ECG and Atrial Contraction Rate Determination

DZX (10 mg/kg) was administered to rats by intraperitoneal injection, and ECG recordings were obtained 90 min thereafter. Heart frequencies were assessed by measuring R-R intervals on standard ECG recordings (limb lead II) in rats anesthetized by the intraperitoneal injection of 12.5 mg/kg xylazine (Q-7833-099; Pisa-Farmacéutica, Mexico City, Mexico) and 80 mg/kg ketamine (Q-7833-028; Pisa-Farmacéutica, Mexico City, Mexico). The HCN4 blocker ivabradine (Procolaran, 5 mg/kg; Servier Laboratories, Mexico City, Mexico) was administered orally. To record the isolated sinus node’s beating rate, rats were injected intraperitoneally with 50 mg/kg sodium pentobarbital. The heart was removed and then placed in Tyrode solution containing 140 mM NaCl, 5.4 mM KCl, 1.2 mM KH_2_PO_4_, 5 mM HEPES, 5.5 mM glucose, 1 mM MgCl_2_, and 1.8 mM CaCl_2_ (Sigma-Aldrich, Burlington, MA, USA). The solution was aerated with 95% O_2_ and 5% CO_2_ to yield a pH of 7.4. A right-atrial preparation containing the sinus node and superior vena cava was dissected and then superfused with Tyrode solution. The right-atrial contraction rate was assessed using an optical video system in which the analog motion signal was digitized and analyzed computationally, as described elsewhere [39]. To analyze the effect of PPC on the atrial contraction rate, we used DZX at a concentration of 100 µM (Tocris Bioscience, Bristol, UK) for up to 90 min in Tyrode solution. DZX was taken from a 0.1 M stock solution. The final DMSO concentration was less than 0.01%. This DMSO concentration was also added to control solutions.

### 4.3. SANC Isolation

SANC isolation was conducted at 35 °C using a protocol described elsewhere [40]. Rats were anesthetized with sodium pentobarbital as described above. The right atrium was dissected, its anterior wall was opened by cutting through the venae cavae to the interatrial septum, and the sinoatrial node was freed by cutting along the crista terminalis. The nodal tissue was cut perpendicular to the crista terminalis to produce three equally sized strips. The strips were washed three times in Tyrode solution supplemented with 1 mg/mL bovine serum albumin, then placed for 30 min in a low-Ca^2+^/Mg^2+^ Tyrode solution with enzymes (pH 6.9) containing 66 µM CaCl_2_, 18.5 mM glucose, 0.55 mg/mL type II collagenase (Worthington Biochemical, Lakewood, NJ, USA), and 0.15 mg/mL type XIV protease (Sigma-Aldrich, Burlington, MA, USA) plus 1.5 mg/mL elastase (Worthington Biochemical, Lakewood, NJ, USA). Then, the tissue was transferred three times to a tube with 2.5 mL Kraftbrühe recovery solution containing 25 mM KCl, 10 mM KH_2_PO_4_, 5 mM HEPES, 20 mM glucose, 20 mM taurine, 100 mM K-glutamate, 10 mM K-aspartate, 2 mM MgSO_4_, 5 mM creatine, 0.5 mM EGTA, and 1 mg/mL bovine serum albumin (pH 7.2). The cells were then dissociated using a fire-polished pipette and gently transferred to a tube containing 2.5 mL Kraftbrühe solution. They were progressively readapted to Ca^2+^ and Na^+^ by the addition of 75 µL and then 160 µL readaptation solution containing 10 mM NaCl and 1.8 mM CaCl_2_, followed by 390 µL, 1.25 mL, and 4.37 mL Tyrode solution. After each solution change, the samples were left to rest for 5 min. The cells were then stored at room temperature until used for patch clamping, RNA isolation, and Western blotting. SANC PPC was performed in Tyrode solution containing DZX (100 µM) for 90 min. At this concentration, DZX is a selective opener of mitochondrial KATP channels [41].

### 4.4. Electrophysiological Analysis

We recorded membrane currents in dissociated elongated and spindle cells from the sinoatrial nodes of adult rats using an Axopatch 200-A amplifier (Axon Instruments, Foster City, CA, USA) and the whole-cell patch-clamp technique, as described previously [4]. To calculate values of membrane capacitance, +10 mV voltage pulses were used. Membrane current recordings lasting 250 ms were digitized at 250 μs intervals with an analog to digital converter (Digidata, Axon Instruments, Foster City, CA, USA) at 16-bit resolution. Square pulses with different amplitude durations were used. The holding potential was −40 mV. The data were analyzed using Sigmaplot ver 10.0, Palo Alto, CA, USA. The bath solution (pH 7.4) contained 140 mM NaCl, 5.4 mM KCl, 1 mM BaCl_2_, 1 mM MnCl_2_, 1.8 mM CaCl_2_, 5.55 mM glucose, and 5 mM HEPES. The pipette solution (pH 7.2) contained 130 mM K-aspartate, 10 mM NaCl, 2 mM MgCl_2_, 5 mM EGTA, 2 mM CaCl_2_, 2 mM Na_2_ATP, 0.1 mM guanosine 5′-triphosphate, 5 mM creatine phosphate, and 10 mM HEPES. To scavenge ROS, we used NAC at a concentration of 2 mM. To block mitoKATP channels, 5-hydroxydecanoate [42] was used (100 µM). Ivabradine (3 μM; Sigma-Aldrich, Burlington, MA, USA) was added 3–5 min before recordings to block HCN4 channel currents. At this concentration, this bradycardic agent partially blocks HCN4 channels [43]. H_2_O_2_ (50 μM) was added 10 min prior to recording. To characterize PPC effects on membrane currents, SANCs were incubated as indicated in the figures and patch-clamped to record whole-cell membrane currents. The experiments were carried out at room temperature (23 °C).

### 4.5. Analysis of HCN4 Channel Currents

To compare current-voltage relation values of If under different experimental conditions with control values, we used the formula:

If = y × gf × (Vm − Ef)(1)

where If represents the current through HCN4 channels, gf is the membrane conductance associated with full HCN4 channel opening (maximal conductance), Vm is the membrane potential during hyperpolarizing pulses, Ef is the reversal potential, and y is a voltage- and time-dependent kinetic parameter that takes values between 0 and 1 and is based on Hodgkin and Huxley’s equations [44]. This approach has been used by others to characterize the kinetic properties of HCN4 channels [45,46].

In the steady state (when t → ∞), y = y∞ and is described by a Botzmann distribution:

y∞ = 1/{1 + exp [(Vm − *V*)/k]} (2)

where *V* represents the membrane potential at which the membrane conductance reaches half-maximum values and k is a measure of the slope. The substitution of Equation (2) into Equation (1) yields the following:

If ∞ = gf × (Vm − Ef)/{1 + exp [(Vm − *V*)/k]} (3)

Equation (3) provides a quantitative description of steady-state values of If as a function of voltage. We used a value of –35 mV for Ef, as proposed elsewhere [46,47] and fitted k and *V* to membrane current values at the ends of hyperpolarizing pulses using a non-linear least-squares method.

### 4.6. Measurement of Ca^2+^ Transients

Intracellular Ca^2+^ signals were measured as described in detail elsewhere [4]. In brief, Fluo-3 acetoxymethyl ester (Molecular Probes-Thermo Fisher, Waltham, MA, USA) was used to monitor changes in the fluorescence of intracellular Ca^2+^ associated with action potentials. SANCs were loaded with approximately 5 mM Fluo-3 acetoxymethyl ester in standard Tyrode solution for 40 min at room temperature. They were then incubated in dye-free solution for >30 min to allow the conversion of the dye to its Ca^2+^-sensitive free acid form. Dye-loaded cells were placed on a laminin-coated coverslip at the bottom of a chamber on the stage of an Optiphot microscope (Nikon, Tokyo, Japan). Fluorescence emitted by stained SANCs, illuminated episcopically with monochromatic light at 485 nm, was passed through a high-pass barrier filter (cut-off wavelength 535 nm) and detected with a low-noise photodiode in a photovoltaic configuration. Analog signals were digitized as described above and sampled every 0.6 ms. The basal fluorescence (F) of the SANCs was recorded, and its mean value during a 60 ms interval prior to the appearance of Ca^2+^ associated with action potentials was used to calculate Ca^2+^ signals as ΔF/F values. To assess the effect of PPC on Ca^2+^ transients, SANCs were DZX-treated using an experimental procedure similar to that used for the electrophysiological experiments.

### 4.7. qRT-PCR Assays

Patch-clamp pipettes filled with Tyrode solution, previously washed with diethyl pyrocarbonate–treated H_2_O, were used to collect four to seven spindle cells. The cells were centrifuged at 70× *g* for 7 min and the supernatant was discarded. A Single Cell-to-CT kit (PN 4458237; Thermo Fisher, Waltham, MA, USA) was used for gene expression analysis. The cells were lysed and cDNA was synthesized following the manufacturer’s instructions. Quantification of mRNA was performed using TaqMan assays (Applied Biosystems, Foster City, CA, USA), Taq-Man Gene Expression Master Mix (4369016), and Rn00572232_m1 primer-probe sets for Hcn4 quantification. Eukaryotic 18S ribosomal RNA (Hs99999901_s1) was used as an internal control with an iCycler iQ (Bio-Rad, Hercules, CA, USA). Quantification was performed using the 2^−∆∆CT^ method [48].

### 4.8. Immunofluorescence

Fresh SANCs were used for confocal microscopic analysis. The cells were attached to laminin-coated coverslips for 2 h at 35 °C, then washed twice with Tyrode solution and incubated for 90 min at 35 °C in Tyrode solution with or without DZX (100 µM). Thereafter, they were washed twice with Tyrode solution, fixed with formaldehyde (4% diluted in phosphate buffer saline (PBS)) for 15 min at room temperature, washed twice again in PBS (1×), and permeabilized with Triton X100 (0.02% in PBS) for 30 min at room temperature. The cells were then washed twice in PBS, blocked for 1 h with 10% normal donkey serum (017-000-121; Jackson ImmunoResearch Laboratories, Inc., West Grove, PA, USA) in PBS, washed twice, and incubated overnight with primary antibody at 4 °C. Then, they were washed three times with PBS and incubated for 1 h with a secondary antibody. The primary and secondary antibodies were monoclonal anti-mouse REST/NRSF (sc-374611; Santa Cruz Biotechnology, Inc., Dallas, TX, USA), polyclonal anti-rabbit HCN4 (APC-052; Alomone Labs, Jerusalem, Israel), monoclonal anti-mouse c-Fos (sc-166940; Santa Cruz Biotechnology, Inc., Dallas, TX, USA), Alexa Fluor 488 anti-mouse (A21202; Thermo Fisher, Waltham, MA, USA); Alexa Fluor 555 anti-rabbit (A31572; Thermo Fisher, Waltham, MA, USA). After incubation, the cells were washed three times using PBS, incubated for 10 min using Hoechst 33342 nuclear dye (1:1000, H3570; Invitrogen, Carlsbad, CA, USA), washed again three times as before, and mounted on coverslips treated with 2 µL Vectashield (Vector Laboratories, Newark, CA, USA).

Confocal microscopy was carried out with argon (488 nm) and helium/neon (543 nm) lasers (TCS-SP8; Leica, Wetzlar, Germany) and an optimized pinhole diameter. Confocal images were obtained as z-stacks of single optical sections, then superimposed to create single images with the LAS AF 2.6.0 build 7268 software, ver. 3.3.0 (Leica, Wetzlar, Germany). They were analyzed using Image J software (ver. 2.7.0; National Institutes of Health, Bethesda, MD, USA). To obtain nuclei–cytoplasm fluorescence ratios, we used the procedure described in detail elsewhere [49].

### 4.9. Western Blotting

Fresh SANCs were incubated in Tyrode solution with or without DZX (100 µM) for 90 min. They were then centrifuged at 300× *g* and 4 °C for 7 min. The supernatant was discarded, and the cells were resuspended in lysis buffer containing 50 mM Tris-Cl, 150 mM NaCl, 0.5% NP-40, 10 mM NaF, and 1 mM Na_3_VO_3_ (pH 7.4). The cells were lysed and processed following the procedure described elsewhere [50]. The antibodies were polyclonal anti-rabbit HCN4 (1:500, 55224-1-AP; Proteintech, Rosemond, IL, USA), monoclonal anti-rabbit REST/NRSF (1:500, A2415; Abclonal Technology, Woburd, MA, USA), and anti-mouse actin (1:500, A3853; Sigma-Aldrich, Burlington, MA, USA). Actin bands were used to normalize HCN4 and REST density values.

### 4.10. Statistical Analysis

The data are expressed as means ± standard errors of the mean. When two groups were compared, paired and independent t tests were used. To compare more than two treatments with a control analysis of variance, the multiple-comparison Dunnett’s test was used. GraphPad Prism ver. 4.0 (GraphPad Software) and Sigma Stat ver. 2.0 were used for statistical analysis. A *p* < 0.05 was considered significant.

## 5. Conclusions

The results of this study indicate that PPC regulates SANC firing activity through ROS effects on ion channels that play a role in the membrane clock via the de novo synthesis of HCN4 channels through a mechanism that possibly involves REST/NRSF and c-Fos. The increase in heart rate induced by DZX may compensate for the decrease in the amplitude of Ca^2+^ transients in ventricular cardiomyocytes during action potentials associated with PPC, leading to normal cardiac output.

## Data Availability

Data are contained within the article and Supplementary Material.

## Supplementary Materials

The following supporting information can be downloaded at: https://www.mdpi.com/article/10.3390/ijms26136062/s1.

## Abbreviations

The following abbreviations are used in this manuscript:

DZX	Diazoxide
IVA	Ivabradine
HCN4	Hyperpolarization-activated cyclic nucleotide potassium channel 4
REST	RE1-silencing transcription factor
NRSF	Neuron-restrictive silencer factor
