# Supplementary material for: Pharmacological Preconditioning with Diazoxide Upregulates HCN4 Channels in the Sinoatrial Node of Adult Rat Cardiomyocytes
Source: Int J Mol Sci. 2025 Jun 24;26(13):6062. doi: 10.3390/ijms26136062 (PMC12249526; doi:10.3390/ijms26136062)
Supplement: Supplementary file 1 [file ijms-26-06062-s001.zip › Figure S1.pdf]

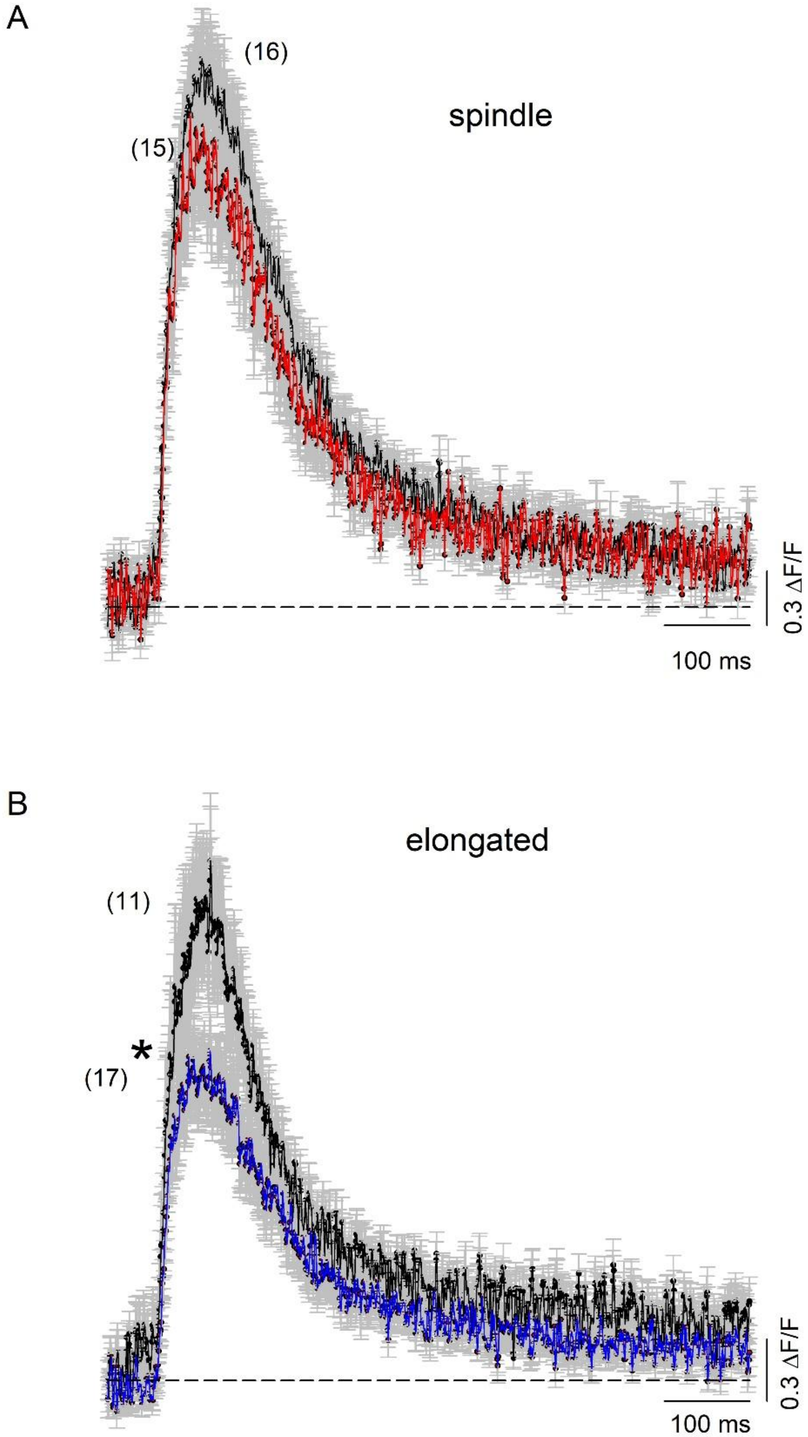

Figure S1. The effect of PPC on Ca^{2+} transients. (**A**), average Fluo-3 fluorescence signals (± SEM) from control spindle cells (black) and after DZX treatment (red), n=15 and n=16, respectively. (**B**), the corresponding results from elongated cells, n=11 (black) and n=17 (blue). Dashed lines indicate basal fluorescence level. * p<0.5.
